# Host- and Species-Dependent Quasispecies Divergence of Severe Acute Respiratory Syndrome Coronavirus-2 in Non-human Primate Models

**DOI:** 10.3389/fmicb.2021.694897

**Published:** 2021-07-09

**Authors:** Eun-Ha Hwang, Hoyin Chung, Green Kim, Hanseul Oh, You Jung An, Philyong Kang, Choong-min Ryu, Jong-Hwan Park, Jungjoo Hong, Bon-Sang Koo

**Affiliations:** ^1^National Primate Research Center, Korea Research Institute of Bioscience and Biotechnology, Cheongju, South Korea; ^2^Laboratory of Animal Medicine, College of Veterinary Medicine, Chonnam National University, Gwangju, South Korea; ^3^Department of Microbiology, College of Natural Sciences, Chungbuk National University, Cheongju, South Korea; ^4^Futuristic Animal Resource Center, Korea Research Institute of Bioscience and Biotechnology, Cheongju, South Korea; ^5^Infectious Disease Research Center, Korea Research Institute of Bioscience and Biotechnology, Daejeon, South Korea

**Keywords:** SARS-CoV-2, non-human primate, single nucleotide variant, genetic variants, single nucleotide polymorphism

## Abstract

Recently, newly emerging variants of severe acute respiratory syndrome coronavirus 2 (SARS-CoV-2) have been continuously reported worldwide. However, the precise evaluation of SARS-CoV-2 microevolution in host is very limited because the exact genetic information of infected virus could not be acquired in human researches. In this report, we performed deep sequencing for seed virus and SARS-CoV-2 isolated in eight cynomolgus and rhesus macaques at 3 days postinoculation and evaluated single-nucleotide polymorphisms (SNPs) in SARS-CoV-2 by variant analysis. A total of 69 single-nucleotide variants (SNVs) were present in the 5′-untranslated region (UTR), 3′-UTR, *ORF1ab*, *S*, *ORF3a*, *ORF8*, and *N* genes of the seed virus passaged in VERO cells. Between those present on the seed virus and those on each SARS-CoV-2 isolated from the lungs of the macaques, a total of 29 variants was identified in 4 coding proteins (ORF1ab, S, ORF3a, and N) and non-coding regions (5′- and 3′-UTR). Variant number was significantly different according to individuals and ranged from 2 to 11. Moreover, the average major frequency variation was identified in six sites between the cynomolgus monkeys and rhesus macaques. As with diverse SNPs in SARS-CoV-2, the values of viral titers in lungs were significantly different according to individuals and species. Our study first revealed that the genomes of SARS-CoV-2 differ according to individuals and species despite infection of the identical virus in non-human primates (NHPs). These results are important for the interpretation of longitudinal studies evaluating the evolution of the SARS-CoV-2 in human beings and development of new diagnostics, vaccine, and therapeutics targeting SARS-CoV-2.

## Introduction

Following the first outbreak of severe acute respiratory syndrome coronavirus 2 (SARS-CoV-2) in a Wuhan seafood market in China in December 2019, the virus has spread rapidly worldwide, resulting in significant severe respiratory disease in humans (Huang et al., 2020; Zhu et al., 2020). Associated clinical features range from asymptomatic infection to mild or severe disease, including fever, fatigue, dry cough, olfactory and taste disorder, acute respiratory distress syndrome, and multiorgan failure (Wu and McGoogan, 2020). As of March 31, 2021, a total of 127,877,462 cases and 2,796,561 deaths were confirmed in 216 countries on the World Health Organization COVID-19 dashboard. Recently, the broad vaccination was initiated in many countries. However, the rapid evolution of this virus causes concern for the efficacy of vaccine.

RNA viruses generally have higher nucleotide substitution rates than other viruses due to the low fidelity of RNA polymerase and absence of postreplication repair systems (Duffy et al., 2008). SARS-CoV-2, a single-stranded, positive-sense, enveloped RNA virus, belongs to the genus *Betacoronavirus* within the family Coronaviridae that contains four other human coronaviruses of SARS-CoV, Middle East respiratory syndrome (MERS) virus, human coronavirus (HCoV)-OC43, and HCoV-HKU1 (Wu and McGoogan, 2020). The SARS-CoV-2 gene is 29,891 nucleotides in length and includes a 5′-untranslated region (UTR), open reading frame (*ORF*) *1a* and *1b*, spike (*S*), envelope (*E*), membrane (*M*), *ORF5*, *ORF7a*, *ORF8*, nucleocapsid (*N*), *ORF10*, and 3′-UTR (Wu et al., 2020). Mutations in SARS-CoV-2 have been continuously reported based on observed changes in the nucleotide or amino acid sequences of the analyzed macromolecules (Korber et al., 2020; Mercatelli and Giorgi, 2020). At present, eight distinct subgroups of SARS-CoV-2 have been reported worldwide in the Global Initiative for Sharing All Influenza Data (GISAID) database. The heterogeneity in nucleotide sequences of recent isolates is 15% greater than those of the first isolates (Benvenuto et al., 2020).

Recently, deep sequencing techniques have revealed that viruses consist of mixed bases at each nucleotide position rather than identical consensus nucleotide sequences in their host. This intrahost ensemble of virus variants has been called viral quasispecies, mutant clouds, and mutant spectra (Domingo and Perales, 2019). These groups of viruses are not simply a collection of various mutants but rather synergize to contribute to the overall characteristics of the population through cooperative interactions (Vignuzzi et al., 2006). Among SARS-CoV-2 patients, the presence of intrahost quasispecies has been identified in each structural and non-structural viral protein (Al Khatib et al., 2020; Karamitros et al., 2020; Shen et al., 2020). This microevolutionary event could affect the infectivity, virulence, and transmissibility of the virus. Minor alleles of the mutant spectrum not only confer resistance to neutralizing antibodies, cytotoxic T cells, and therapeutics but also change the immune response and virion stability (Domingo and Perales, 2019). Therefore, viral quasispecies can change the tissue tropism, virulence, infectivity, and transmissibility of viruses, challenging the development of vaccines, therapeutics, and diagnostics.

A non-human primate (NHP) model is one of the preclinical animal models closest to humans based on phylogenetic, physiological, anatomical, and immunological aspects. For this reason, NHP models have been continuously used for the study of human infectious disease, especially SARS-CoV-2 (Gardner and Luciw, 2008; Koo et al., 2020). Viral quasispecies have been reported previously in humans (Karamitros et al., 2020; Shen et al., 2020). However, research on viral quasispecies in humans has limitations with regard to single-nucleotide polymorphism (SNP) analysis due to a lack of knowledge of the exact genetic information of the seed virus. In this report, we performed SNP and variant analyses using two NHP models infected with identical strains of SARS-CoV-2 with the goal of furthering the comprehensive understanding of evolution of this deadly virus.

## Materials and Methods

### Virus

The SARS-CoV-2 strain (registration number 43326) used in this study was isolated from a Korean patient traveling to China (the National Culture Collection for Pathogens) (Cheongju, Korea). Following plaque purification of SARS-CoV-2, this virus was cultivated twice in VERO cells with Dulbecco’s minimum essential medium (Welgene Inc., Daegu, Korea) containing 2% fetal bovine serum and 1% penicillin (10,000 IU/ml)/streptomycin (10,000 IU/ml) (Gibco, NY, United States) at 37°C in a 5% CO_2_ incubator. At 3 days postinoculation (dpi), the cell supernatant was collected, centrifuged at 3,000 *g* for 10 min, and stored at −80°C. Virus titration was performed using VERO cells, and the 50% tissue culture infectious dose 50/ml (TCID_50_/ml) was calculated according to the Reed–Muench method (Reed and Muench, 1938).

### Animal Experiments

A total of eight, 3- to 6-year-old, healthy, Cambodian-origin cynomolgus (*Macaca fascicularis*) and Chinese-origin rhesus (*Macaca mulatta*) macaques were equally selected and reared in indoor cages at the National Primate Research Center (Korea Research Institute of Bioscience and Biotechnology) (Table 1). All animals were fed fresh fruits and commercial monkey feed during the experimental procedures (Teklad, Global 20% Protein Primate Diet). They were transferred and reared in negative-pressure, NHP specific isolators in an Animal Biosafety Level 3 (ABSL-3) facility (Three-Shine Inc., Seoul, Korea). After 1 week, these animals were inoculated under anesthesia with 12.5 ml (2.1 × 10^6^ TCID_50_/ml) of the same batch of virus via oral (5 ml), intratracheal (4 ml), nasal (1 ml), conjunctival (0.5 ml), and intravenous (2 ml) routes, as previously reported (Koo et al., 2020). At 3 dpi, all six lobes of the lungs of all animals were aseptically collected, homogenized with sterilized 10-fold (w/v) phosphate buffered solution (pH 7.4), and filtered with a 0.2-μm pore size syringe filter for virus isolation. The presence of viable viruses from lung tissues was examined by virus isolation using VERO cells, and the TCID_50_/ml was calculated according to the Reed–Muench method (Reed and Muench, 1938). Hematological analysis was performed from ethylenediaminetetraacetic acid (EDTA) blood samples with an auto-hematology analyzer (Mindray Inc., Shenzhen, China).

**TABLE 1:** Information on the animals and SARS-CoV-2 used in this report.

Animal	Species	Birth date	Sex	Body weight (kg)	Tissues	Viral loads	
						TCID_50_/ml	Copies/ml
CM1	*Macaca fascicularis*	July 30, 2014	Male	5.05	Lung	9.3 × 10^2^	2.7 × 10^8^
CM2	*Macaca fascicularis*	September 7, 2013	Male	4.57	Lung	4.9 × 10^2^	1.3 × 10^8^
CM3	*Macaca fascicularis*	December 29, 2014	Female	3.2	Lung	4.3 × 10^4^	1.7 × 10^9^
CM4	*Macaca fascicularis*	September 29, 2013	Female	3.68	Lung	6.3 × 10^4^	1.3 × 10^9^
RM1	*Macaca mulatta*	March 11, 2015	Female	4.89	Lung	2.6 × 10^3^	1.6 × 10^8^
RM2	*Macaca mulatta*	January 16, 2015	Female	3.95	Lung	1.6 × 10^4^	1.3 × 10^8^
RM3	*Macaca mulatta*	October 6, 2014	Male	4.5	Lung	5.4 × 10^2^	3.0 × 10^7^
RM4	*Macaca mulatta*	June 6, 2015	Male	4.954	Lung	4.3 × 10^2^	5.9 × 10^7^

### Deep Sequencing and Variant Analysis

The whole genome sequence of SARS-CoV-2 was sequenced using the BTSeq^TM^ SARS-CoV-2 WGS Service (Celemics Inc., Seoul, Korea). Reverse transcriptase was used to synthesize complementary DNA (cDNA). The viral cDNA library was amplified with primers designed based on a published SARS-CoV-2 sequence database (data not shown). Using the amplified DNA, a DNA library was constructed through DNA fragmentation, end repair, adaptor ligation, and PCR amplification for next-generation sequencing (NGS). The DNA library was purified with CeleMag DNA Clean-up Beads (Celemics Inc.). Quantification was conducted using an Agilent 2200 TapeStation (Agilent Technologies, Sta. Clara, CA, United States). After quantification, NGS was performed using a MiSeq next-generation sequencer with 150PE (Illumina, San Diego, CA, United States). Sequence data were imported as fastq files and edited using the CLC Genomics Workbench 11.0.1. All low quality (<0.05), ambiguous (0), short (under 64 nucleotides), and adapter sequences were removed in the trimming procedure. Trimmed reads were mapped to a reference strain (GenBank number MN908947.3). Intrahost variant analysis of SARS-CoV-2 was performed against the genome of seed virus using the basic variant analysis in the CLC Genomics Workbench 11.0.1. Minor alleles were identified based on criteria of >50 read depth, >5% minor allele frequency, and >5 minor allele count. The frequency difference of the major and minor alleles was statistically verified using Fisher’s exact test, with a *p* ≤ 0.05 considered statistically significant.

### Ethical Approval

All experimental procedures were performed in an ABL3 facility and approved by the Institutional Animal Care and Use Committee of the Korea Research Institute of Bioscience and Biotechnology (permit number KRIBB-AEC-20064).

## Results

### Sequencing Data

**FIGURE 1:**
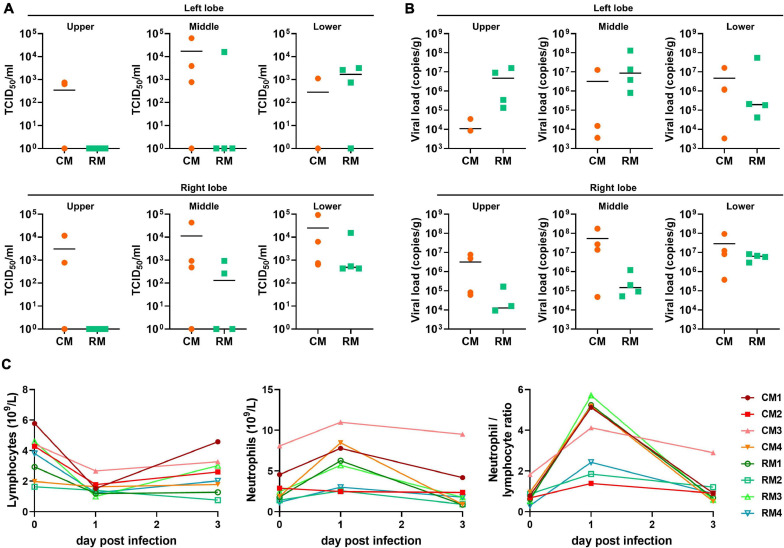
Viral loads in lower respiratory samples and hematological values in cynomolgus and rhesus macaques inoculated with SARS-CoV-2. At 3 dpi, the six lobes of lungs were collected; viral loads in all lobes were determined by **(A)** TCID_50_ and **(B)** qRT-PCR. The bars indicate the median value of viral loads in each animal. **(C)** The values of hematology were evaluated at 1 and 3 dpi.

**FIGURE 2:**
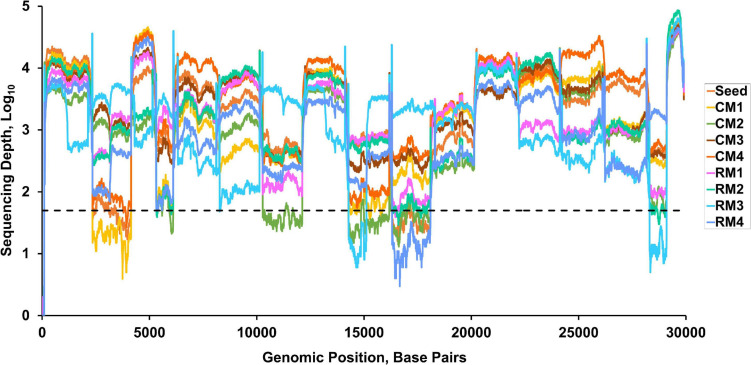
Read depth across the whole genome of SARS-CoV-2. Dashed line indicates a read depth of 50.

Viable SARS-CoV-2 was successfully isolated in the lungs of all monkeys at 3 dpi. Virus titers in each animal were successfully determined expressed as TCID_50_ and viral copy load (Figure 1). One of the six lobes with the highest viral titers in each animal was selected for further analysis based on TCID_50_ and viral copy number (Table 1). In addition, hematological values, body weight, rectal temperature, and respiratory rates of animals were described in Figure 1 and Supplementary Table 1. Using deep sequencing, an average of 2,595,447 reads per sample were obtained after quality control. After assembly mapping these data to the reference genome of SARS-CoV-2 (NCBI accession number MN908947), the average number of mapped reads per sample was 1,294,312. The mean read depth was 4,915×, and the breadth of coverage rate was 99.74% of the 29,903 nucleotides in the SARS-CoV-2 genome (Supplementary Table 2). Similar fluctuations in the pattern of read depth at each nucleotide position were identified among samples (Figure 2). All sequence data were submitted and deposited in the GenBank Sequence Read Archive (BioProject number PRJNA688005).

### Intrahost Variant Analysis

**FIGURE 3:**
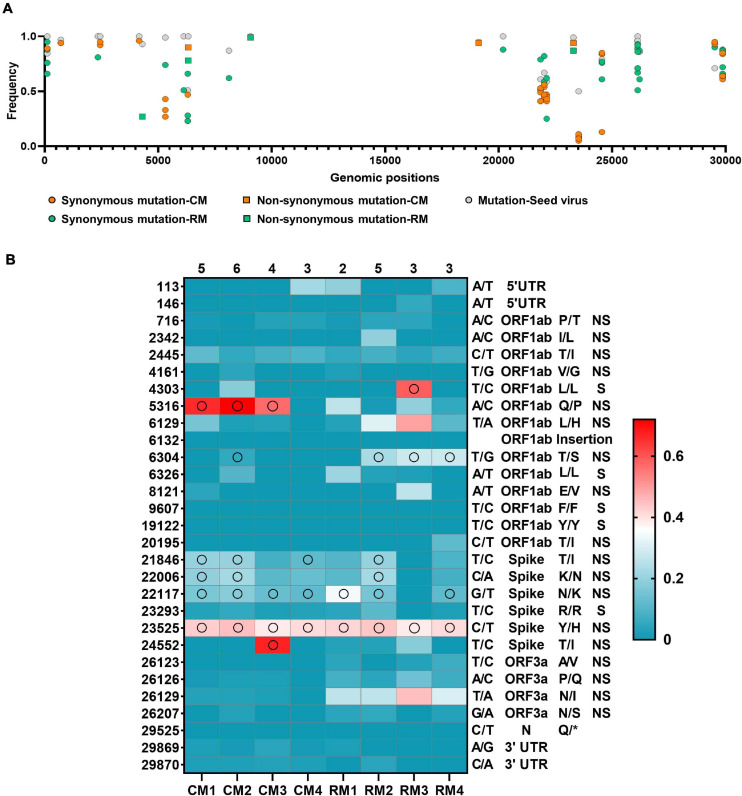
Mutation frequency in the SARS-CoV-2. **(A)** Frequency variant profiles within the whole SARS-CoV-2 genome. The mutations of major allele frequency of seed virus were described at the consensus level. **(B)** Changes in frequency rates of corresponding alleles at each position in SARS-CoV-2 isolates compared to the reference alleles of the seed virus. Reference allele/substitute allele at the nucleotide position, gene name, amino acid variation (reference/substitute), and mutation type (non-synonymous, synonymous) are shown to the right of the heat map. Open circles represent consensus variants at this position. The total number of consensus changes in each sample is shown above it.

**FIGURE 4:**
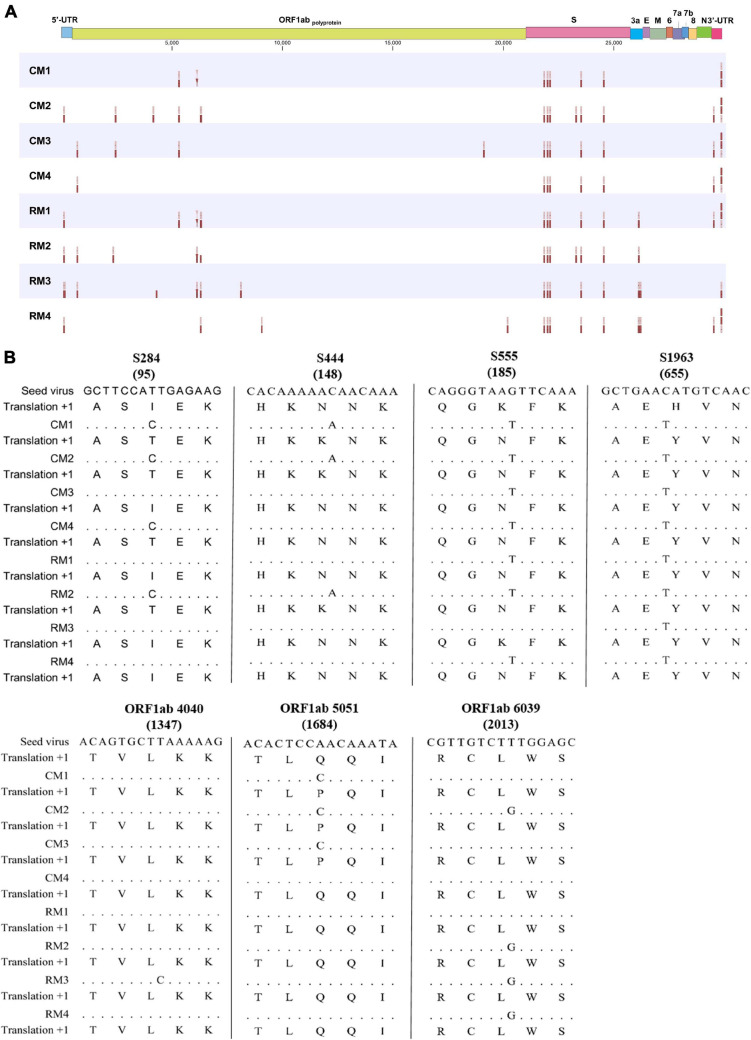
Variant profiles within the whole SARS-CoV-2 genome. **(A)** Interhost variants in single nucleotide variants and **(B)** consensus sequences of the seed virus and SARS-CoV-2 isolates. The number in parenthesis indicates amino acid position.

A total of 69 SNVs were identified in the 5′- and 3′-UTRs and the *ORF1ab*, *S*, *ORF3a*, *ORF8*, and *N* genes of the seed virus (Supplementary Table 3). Frequency mutation of major alleles in each isolate was evaluated in the different sites between isolates and seed virus at the consensus level (Figure 3A). Non-synonymous mutations were mainly observed in the *S* gene of the seed virus. Different consensus sequences were identified in three sites of the *ORF1ab* and four sites of the *S* genes between the seed and isolated SARS-CoV-2 viruses (Figure 3B). All four mutation sites in the *S* gene were non-synonymous substitutions, whereas two of three substitutions in *non-structural protein* (*NSP*) 3 within *ORF1ab* corresponded to non-synonymous substitutions (Figure 3). On variant analysis based on >5% minor allele frequency and >5 minor allele count, a total of 29 variants was identified in 4 coding genes (*ORF1ab*, *S*, *ORF3a*, and *N*) and non-coding regions (5′- and 3′-UTR). The most common variant type (28/29) was SNV, with one insertion site (Figure 4A). Among the analyzed genes, the number of identified SNVs was highest in *ORF1ab* (14/28), followed by *S* (6/28), *ORF3a* (4/28), 5′-UTR (2/28), 3′-UTR (2/28), and *N* (1/28) (Figure 3B). These SNVs in viral coding genes mainly corresponded to non-synonymous mutations (18/24, 75.0%) rather than synonymous (5/24, 20.8%) or stop mutations (1/24, 4.2%). The rate of non-synonymous mutations was highest in *ORF3a* (4/4, 100%), followed by *S* (5/6, 83.3%) and *ORF1ab* (9/13, 69.2%). The stop codon was only observed in the *N* gene.

### Interhost Variant Analysis

Interhost distinct SNVs and consensus sequences were identified among the genomes of SARS-CoV-2 isolated in individual macaques, even though the same seed virus replicated in the lungs of these monkeys for only 3 days. Different consensus sequences were identified in three sites of the *ORF1ab* and four sites of the *S* genes between the seed and isolated SARS-CoV-2 viruses (Figure 4B). All four mutation sites in the *S* gene were non-synonymous substitutions, whereas two of three substitutions in *NSP* 3 within *ORF1ab* corresponded to non-synonymous substitutions. A total of seven different consensus sequences at six sites were identified among some, but not all, monkeys. Variant analysis comparing isolates to the seed virus revealed that the number of SNVs ranged from 9 to 16 among the individual monkeys (Figure 4). The number of statistically different variant sites ranged from 2 to 11 by monkey. These differences in SNVs were mainly identified in the *ORF1ab* gene. The differences at six sites (nucleotide positions 5316, 6129, 6304, 26123, 26129, and 26207) were statistically verified based on average values of major allele frequency variations between the two macaque species (Supplementary Table 4). Moreover, the site numbers of frequency differences in SARS-CoV-2 in rhesus monkeys were more frequent compared to those in cynomolgus monkeys in variant analysis (Supplementary Table 5).

## Discussion

SARS-CoV-2 infection has been the cause of the most prevalent and devastating respiratory disease worldwide after the first outbreak in late 2019. Recently, newly emerging variants have been continuously reported to cause significantly higher morbidity and mortality than previous viruses despite enforcement of strict biosecurity policies in many countries. Therefore, there is an increasingly urgent need for vaccines and therapeutics against SARS-CoV-2. The precise evaluation of its viral genomic evolution should be conducted for the development of effective vaccines and therapies against SARS-CoV-2. Macaque models of cynomolgus and rhesus monkeys were successfully constructed via the combined routes, which have been reported as possible natural routes of transmission in human (Li H. et al., 2020; Lu et al., 2020). In this report, we characterized the genomic mutations of SARS-CoV-2 in two macaque species infected with the same seed virus by deep analysis.

Seventy-six SNVs were identified in the 5′ and 3′-UTRs and genes that encode for four viral proteins (ORF1ab, S, ORF3a, and N) of the seed virus, which was serially passaged in VERO cells. The highest number of SNVs was observed in *S* gene. The S protein of SARS-CoV-2 is a well-known surface glycoprotein and is closely associated with its antigenicity, pathogenicity, and tissue tropism. Most SNVs in the *S* gene corresponded to non-synonymous mutations, indicating the possibility of a changed phenotype. The second highest number of SNVs was found in *ORF1ab*, a gene known to encode for a replicase/transcriptase involved in the virulence, virus–cell interaction, virus–host response, as well as replication, of SARS-CoV-2 (Graham et al., 2008). An *ORF3a* with high mutation rate could induce changes in virulence, infectivity, ion channel formation, and virus release; however, the SNVs identified in this report were not found in the functional domains of ORF3a (Hassan et al., 2020; Issa et al., 2020). Overall, the mutation sites we identified within the *ORF1ab*, *S*, and *ORF3a* genes seem to be related to increased fitness in VERO cells by serial passage and inversely induced attenuated pathogenicity and changed antigenicity for an original host, human (Hanley, 2011). Therefore, these sites should be evaluated for their exact role in pathogenicity and antigenicity of SARS-CoV-2 in humans, as they may represent a new target for development of novel vaccines and therapeutics.

After identical seed SARS-CoV-2 were replicated in the lungs of monkeys for 3 days, a total of 29 variant sites were identified between the seed virus and SARS-CoV-2 isolates. Based on the consensus sequence, only seven sites of allele frequency changes in the genome were determined between the seed virus and the isolates from each individual macaque. Distinct results obtained with two evaluation methods indicated that precise SNV analysis using deep sequencing should be conducted for exact evaluation of viral evolution. The nucleotide substitution rates of SARS-CoV-2 have proven to be relatively fast compared to other viruses (Duffy et al., 2008; Li X. et al., 2020). A total of eight genotypes with distinct phenotypes have been reported worldwide in the GISAID database within approximately 1 year of the first outbreak (Elbe and Buckland-Merrett, 2017). The presence and evolution of these genetic mutations in SARS-CoV-2 was evaluated using consensus sequences. Our results indicate that more complicated and faster viral evolution could proceed in host monkeys, as expected based on previous reports. Importantly, the allele frequency changes with non-synonymous mutations were most frequently observed in the *S* gene, which could lead to significant possible changes in antigenicity, virulence, and transmissibility of the virus. Almost all changes in *ORF1ab* occurred in the Nsp3 region, which codes for the papain-like protease PLpro that regulates SARS-CoV-2 viral spread and innate immunity (Shin et al., 2020). In this report, the grades of lymphopenia as a cardinal marker for clinical severity were significant differentiated between cynomolgus and rhesus macaques at 3 dpi (*p* = 0.02, data not shown). Distinct frequency allele change between two species was identified in only ORF 3a (nucleotide position 26129). However, viral loads and other hematological pathological indicators (thrombocytopenia, neutrophilia, and neutrophil/lymphocyte ratio) were identified variable according to each animal, and the link between specific genetic mutation and phenotypic changes could not be determined (Supplementary Table 1). However, this intrahost ensemble of virus variants could contribute to the overall phenotypes of the population through cooperative interactions (Vignuzzi et al., 2006). In conclusion, non-synonymous mutations in the regions of genomes associated with changes in the antigenicity and virulence of SARS-CoV-2 occurred in hosts after a very short replication period. This phenomenon could help explain the pattern of reinfection with newly emerging genotypes that has been reported in human patients (Tillett et al., 2020; To et al., 2020).

A key finding of this report is that the same seed virus differentially evolved in eight individual monkeys despite the short replication time of 3 days (Figure 3). Different frequencies of variation were identified in the genomes of SARS-CoV-2 among the individual macaques; the number and position of the variation sites were different among individuals. Hypervariable regions were identified within the partial regions of genome of ORF1ab and S proteins. The 11 variations sites in RM3 were statistically identified, whereas only 2 sites were determined in CM4. These variable SARS-CoV-2 microevolution could affect distinct virus replication pattern in each macaque (Figure 1). The absolute number of SNV sites and pathogenicity have been reported to decrease due to bottleneck transmission among hosts (Gutiérrez et al., 2012). However, distinct viral microevolution that occurs within individual hosts could lead to new genotypes after interhost transmission. Distinct simplified SNVs were observed in a contact group after interhost bottleneck transmission of influenza virus (Varble et al., 2014). Additionally, differences in average allele frequency between the two macaque species was verified in six sites of SARS-CoV-2. In humans, differences in virulence according to race and ethnicity have been reported in the United States (Vahidy et al., 2020). Our results suggest that host genetics could induce differences in viral evolution of SARS-CoV-2, which could in turn lead to phenotypic differences, including virulence, in humans.

Recent analyses of SNVs in SARS-CoV-2 isolated from human patients have been reported (Karamitros et al., 2020; Shen et al., 2020). However, given the lack of information on the seed virus when SARS-CoV-2 is isolated from human patients, there remains difficulty in evaluating its exact viral evolution in humans. In this report, the high intrahost, interhost, and interspecies genomic variability of SARS-CoV-2 following a short replication period were verified using two species of macaques. Further researches should be conducted to determine the impact of each viral variant on clinical outcomes in NHPs. This information should be valuable not only for understanding the viral evolution of SARS-CoV-2 in hosts but also for the development of new diagnostics, vaccines, and therapeutics for this fatal disease.

## Data Availability Statement

The datasets presented in this study can be found in online repositories. The names of the repository/repositories and accession number(s) can be found in the article/Supplementary Material.

## Ethics Statement

The animal study was reviewed and approved by the Institutional Animal Care and Use Committee of the Korea Research Institute of Bioscience and Biotechnology.

## Author Contributions

E-HH and B-SK performed the experiments and wrote the manuscript. HC, GK, HO, YA, PK, C-MR, and J-HP performed the data analysis. JH and B-SK designed the study and supervised the experiments. All authors read and approved the final version of the manuscript.

## Conflict of Interest

The authors declare that the research was conducted in the absence of any commercial or financial relationships that could be construed as a potential conflict of interest.
