# Supplementary material for: Host- and Species-Dependent Quasispecies Divergence of Severe Acute Respiratory Syndrome Coronavirus-2 in Non-human Primate Models
Source: Front Microbiol. 2021 Jul 9;12:694897. doi: 10.3389/fmicb.2021.694897 (PMC8299785; doi:10.3389/fmicb.2021.694897)
Supplement: Supplementary file 1 [file Data_Sheet_1.zip › Table 1 (8).DOCX]

Supplementary Material

# Supporting information captions

Table S1. Hematologic values and vital signs of cynomolgus and rhesus monkeys.

Table S2. Summary of NGS data in the SARS-CoV-2 genome of seed virus and isolates.

Table S3. SNVs in the seed virus.

Table S4. Differences in average frequency changes between the SARS-CoV-2 seed virus and isolates of cynomolgus and rhesus monkeys.

Table S5. Average allele frequency changes between SARS-CoV-2 isolates and the seed virus.
